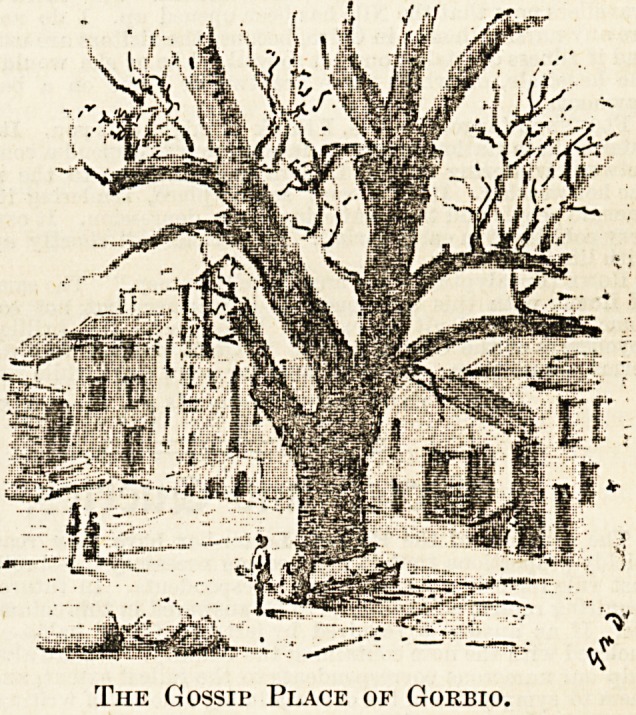# "The Hospital" Nursing Mirror

**Published:** 1899-04-29

**Authors:** 


"The Hospital" Nursing Mirror.

Being the Nursing Section of "The Hospital."

[Contributions for this Section of "The Hospital" should be addressed to the Editor, The Hospital, 28 & 29, Southampton Street, Strand, London, W.C., and should have the word "Nursing" plainly written in left-hand top corner of the envelope.]

### Notes on News from the Nursing World.

### THE DECORATION OF THE ROYAL RED CROSS.

We warmly congratulate the five ladies who have received from the Queen the much valued decoration of the Royal Red Cross. Miss Catharine Sarah Mowbray, late of the Indian Nursing Service; Miss Mary Ellen Barker, of the Indian Nursing Service; and Miss Theresa McGrath owe the bestowal of this coveted honour to the services rendered by them in connection with the nursing of the sick and wounded during the late operations on the Punjab Frontier; while it has been awarded to Miss Sarah Lucy Wildshaw and Miss Mary Ellen Harper, of the Army Nursing Service, in recognition of their services in tending the sick and wounded in Egypt in connection with the recent operations in the Soudan. In each instance the devotion of the nurses to their duty is well-known, and it is fitting that the announcement of the well-earned decoration should have been made at the same time as that of the bestowal of the Victoria Cross and medals on gallant soldiers. The heroism of women in the wards is not less than that of men on the field of battle.

### TROPICAL DISEASES.

At the opening of the Liverpool School for the Study of Tropical Diseases on Saturday Mr. W. Adamson, the President of the Royal Southern Hospital—which has been specially set apart for the purposes of the school—said that the committee "hoped to train nurses who would go abroad properly qualified and having a special knowledge of tropical diseases." The need for this has long been apparent. At present a nurse sent to a tropical climate not only does not understand the class of disease she is likely to have to treat, nor the properties of the drugs she will have to administer, but she has necessarily an insufficient idea of the preventive measures she ought to adopt when she finds that her own health is becoming unsatisfactory. It has just been shown in the case of the nurses who have returned from the Niger that white women of sound constitution and careful habits can live for a year where it was originally supposed none but natives could exist. When it is practicable to obtain the advantages of fuller study and greater experience, which will shortly be possible in both London and Liverpool, the risks of nurses undertaking service in the tropics will be distinctly diminished, while their capacity of ministering to their fellow creatures will be considerably augmented.

### NEW NURSES' HOME AT PEMBROKE DOCK.

Lady Meyrick opened the new nurses' home at Pembroke Dock last week. It is named the Victoria, and with the additional wards forms the local memorial of the Queen's Diamond Jubilee. There are two wards, each containing six beds, one for men and the other for women; and these and the site upon which the whole block of buildings has been erected are the gifts of Sir Thomas Meyrick. The nurses' quarters have accommodation for four nurses and the caretakers, the cost of which has been defrayed by public subscription. The furniture has been given by various residents in the locality. The new buildings are in the Elizabethan Gothic style, and have cost altogether £1,200.

### CONSUMPTIVES AND NATAL.

Nurses who are often consulted by the friends of consumptive patients as to the question of change of climate will do well to note what the Agent-General for Natal has to say on the subject. Under certain conditions, he affirms, the advice to send male patients to Natal is good. This applies to cases where the disease is only threatened, or has become but slightly developed, and there is a reasonable prospect of recovery, providing also that the patients have sufficient means of their own for their support. But as there is no provision for the treatment of consumptives otherwise than in the hospitals, as the expenses in cases of sickness are greatly in excess of the expenses at home, and as the private benevolence of the small European community is apt to be overstrained, Mr. Peace wisely insists that "it is extremely undesirable that invalids in the last stage of consumption should be sent out there to die or to be sent back in a dying condition." The caution of the Agent-General has been rendered essential by the too frequent practice of dispatching consumptives indiscriminately to South Africa.

### THE NURSES OF LEICESTER.

The annual meeting of the Institution of Trained Nurses for the Town and County of Leicester was held last week, the Mayor (Alderman Clifton) presiding. There are two branches of the Institute—a private staff, which is maintained at a high level of efficiency, and a district staff of 11 nurses. The former is prosperous. The nurses have a comfortable home, good salaries, bonuses, and convalescent and gratuitous aid. The subscriptions to the latter are much too small, considering that about £1,000 a year is needed to work the districts satisfactorily. The necessity of forming a fourth district is pressing, and there is some idea of utilising the services of lady volunteers, who have been trained by the "First Aid" courses of instruction, to meet in some measure the growing requirements of the town. Thanks to special efforts made for the purpose, the deficit in the accounts of the District branch during the year has been covered.

### THE PALESTINE AND LEBANON NURSES' MISSION.

The annual meeting of this mission was held on Friday at three p.m. in the Parlour of Exeter Hall. The chair was taken by the Rev. F. Paynter, who was supported by the Rev. R. Douglas, M.A. Miss Wordsworth Smith (the foundress of the mission) was also present. A satisfactory report was read, in which the good work done in the cottage hospital during the year was especially mentioned. An appeal was made for funds (£300) to enable the mission to build a new out-patient department so that the present one may be turned into wards for men. Three ladies who have worked in Baaklem for some years gave interesting addresses. Miss Kitching, who is about to return, spoke of the work in general. Sister Alice Eliot gave an account of the progress and expansion of the medical work since 1891. The spiritual welfare of the poor ignorant Druses was the text of Miss Ward's address. The meeting was well attended, and the audience appeared interested and attentive.

### PASSMORE EDWARDS HOSPITAL FOR WILLESDEN.

A very successful "Café Chantant" in aid of the children's ward of the Passmore Edwards Hospital at Willesden was recently held at the Portman Rooms. The charitably-disposed people who went there from a sense of duty were well rewarded; for, thanks to the indefatigable exertions of the promoters of the entertainment, and the generosity of the professionals and others, an excellent programme of songs, recitations, &c., was carried out under the able direction of Mrs. St. Hill during the afternoon. The tea-room was well patronised, and palmistry was as much in vogue as ever. Miss Frost, the matron, has been devoting all her energies to the cause during the weeks her wards have been in the hands of the builder; for Willesden in the future will boast a hospital of 30 beds, and considerable alterations and additions are being completed. The money realised by the entertainment is likely to prove a substantial help.

### THE LAW AS TO PALMISTRY.

It will be noticed that palmistry was one of the features of the Willesden Café Chantant, and no doubt a considerable sum was realised by the fees of persons who had their "fortunes told." But perhaps it is worth while on this point to print a word of warning. Palmists are still advertising for pupils, and announcing the reception of clients. But the judgment of the Court of Queen's Bench the other day was that palmistry is illegal, and though the Home Secretary on one occasion declared it, "apart from deception," to be permissible, there is no doubt that ladies who receive fees in the cause of charity for their revelations, run a risk of being proceeded against. We are afraid that even the plea that a hard-working nurse was the fortune-teller would not, in that case, suffice to secure immunity from penalty.

### THE CONVALESCENT HOME FOR THE LONDON HOSPITAL.

Although the financial results of the ball last week are substantial enough to enable the authorities of the London Hospital to open the surgical convalescent home at Tankerton, near Whitstable, in May, it must not be supposed that provision is ensured for the ordinary convalescents who require a change of air. The Home in Kent, which will have every advantage so far as situation is concerned, will merely supply accommodation for the comparatively small number of patients with open wounds who require sea air and surgical nursing, and who are not received in ordinary convalescent homes.

### DISTINCTIONS AND DIFFERENCES.

A contemporary has lately directed attention to the grades of nurses employed in the workhouse infirmaries. Drawing a parallel between the varying values of medical degrees, it asks whether, "These Guardians who talk so vigorously about always giving the poor first-class nursing insist also that their officers shall have taken the London M.D.?" It also contends that a woman qualified to nurse a person suffering from senile decay should not be called an inferior nurse, because she is not qualified to take charge of a difficult surgical case. "For instance," it points out, "a plain needlewoman is not an inferior dresssmaker, plain needlework and dressmaking being distinct branches of the seamstress's art." The difficulty is, of course, that some cases need the highly skilled nurse, even if the majority would be equally well served by the trained attendant; and this reminds us of the question that many are asking, namely, "Why should not an acute case be removed to a hospital, or a central infirmary?" It would certainly be more economical; it would bring acute cases together in sufficiently large numbers to keep the skilled nurses fully employed; and it would relieve the Guardians of the task of obtaining trained nurses to stay in workhouses.

### THE FREE HOME FOR THE DYING.

The concert on May 16th at Grosvenor House will bring before the public once more a charity that appeals to the sympathies of all. We refer to the Free Home for the Dying, at The Chase, Clapham. The sole qualification for a candidate is that he or she is supposed to be dying. No money is asked from patients or their friends; no distinction is made as regards nationality, sex, age, or religious faith; everything that is possible is done to comfort the sorrowful, and to alleviate the suffering, inmates. Last year the record of work shows an increase; 63 patients were admitted, 38 died, 24 were discharged, and 11 still remain. Two explanations are given as to the number discharged; some cases improve, and others are of a long chronic nature, and therefore unsuitable. Out of an income of £1,435, £250 was transferred to the building account. An effort is being made to provide a permanent home, for which funds are badly wanted.

### "OUR NURSING SISTERS."

Through the courtesy of the editor of the *Englishwoman* we have been favoured with an advance proof of an admirable article on "Our Nursing Sisters," which appears in the May number of the magazine. The article is copiously illustrated. Pictures of several wards of the London Hospital are given; and not less interesting than these are the portraits of several nurses. Many useful particulars are supplied respecting the staffs of various institutions, the uniforms, the training, and the general arrangements. The portrait of Sir Henry Burdett, as founder of the Royal National Pension Fund for Nurses, is given. The Pension Fund is now the largest thrift organisation for women in the world.

### SHORT ITEMS.

The nurses who sailed in the "Sokoto" for the Niger on Saturday were Miss E. K. Nevill, from Charing Cross Hospital; Miss Isabel Carter, of the Registered Nurses' Society; and Miss Ward, also from London. They will proceed direct to Jebba.—Her Excellency Lady Ridgeway, wife of the Governor, has become the patroness of the Ceylon Nurses' Association.—The staff of the Naval Nursing Service numbers 28 sisters. They work in the Naval Hospital at Haslar, Stonehouse (Plymouth), Chatham, and Malta. A recent move has taken place at Malta, where there are four sisters, Miss Moore having returned to England for duty on completion of her three years abroad, her place being filled by Miss Beattie from Plymouth.

### Antiseptics and Operations.

A Lecture delivered to the Nurses of the Victoria Hospital, Hull, by ALFRED PARKIN, M.S., M.D. (Lond.), Senior Surgeon to the Hospital.

(*Continued from page* 48.)

THE most important means of overcoming germs is to be as clean as possible. If we have dirty faces it means we have a huge quantity of dirt and grease on our faces, and dirt is another name for micro-organisms. The hair, especially in women, is a means of catching dirt and germs, and you no doubt have often seen children who come as out-patients whose hair is not clean, and have had to get rid of thousands of not only germs, but animals, which thrive on the human head. To keep the number of germs as low as possible, great cleanliness is necessary.

The first point in the prevention of germ infection is cleanliness. By that I do not mean washing yourself in the morning, and then if you have not time at night, saying, "Oh! I will leave it till the morning." Not only your face, not only your hands must be clean, but, what is equally as important, your dress, because your dress can catch up any number of germs, and that is why in hospital a nurse must wear whatever is washable. For instance, a sealskin jacket would not be a suitable thing to wear in a hospital; it would be equally useless and dangerous, for it could not be washed. Cleanliness is a big term, and covers not only personal cleanliness, but everything with which you come in contact. And in hospital life you will find you have to be a great deal more careful than you have to be even in private life.

I cannot myself conceive anything more filthy than to go from examining one patient direct to another without washing one's hands. It is repugnant to my feelings, and I would not have a doctor who did so. I once knew an old gentleman who said to me, "Do you know, I shall really have to give up my doctor. He has some ugly pimples on his face, and while he is talking to me he keeps touching these pimples, and then he comes and examines my mouth." And I consider that old gentleman was quite right. I think you will notice that I never go from one patient to another without washing my hands. I know it is a bit of trouble, and gives trouble to others, but it is the right thing to do. If you go, say, from a case of erysipelas on to another case, you can most easily give erysipelas to the second patient, and if he dies his death is on your hands, or ought to be on your mind. So cleanliness, as you will see, is a most important thing, for these germs cannot live in soap and water. And do not forget that these germs are extremely small and can get into tiny cracks where other things cannot. That is the reason why you should keep the finger nails cut short and take care no dirt gets in them, because, as I was explaining before, it is so easy for you, with germs on your fingers or hands, to come and handle sponges or dressings, and so directly poison any patient's wound, and I have not the least doubt that many patients' lives have been sacrificed by the want of understanding, both in the past and in the present, of what germs are and how they affect people.

Now, after cleanliness, *i.e.*, the free use of soap and water and nail brushes, we come to the things which kill germs, and these are called antiseptics. "Antiseptic" means something that will prevent sepsis or poisoning, but it is easier for you to understand that "antiseptic" means something that will kill germs, or, at any rate, will prevent their growing. It comes to much the same thing for you to kill a thing or prevent it growing. One of the very best antiseptics is boiling water, but this cannot be used directly on a patient on account of its scalding effects; it is, however, of great use in cleaning instruments and in the preparation of towels and sponges. Please remember that germs cannot live in boiling water.

There are many antiseptics, and they are put into use in all sorts of ways. You have been entrusted with the use of carbolic and perchloride of mercury, and it is just as well you should know the strength at which these are used, because you might be told at some time to wash yourself in carbolic, and if you used it too strong you would do yourself harm, and if too weak you would do no good at all. Carbolic is generally used 1 in 40—that is, 1 oz. of carbolic to 40 ozs. of water. Perchloride of mercury is used of a very different strength. What we use upstairs is either 1 in 1,000, or 1 in 2,000. You see if you made a mistake and used carbolic 1 in 1,000 you would find you did absolutely no good, and if you used perchloride of mercury in the proportions of 1 in 40 you would kill your patient in two or three days. Commit these figures to memory, for some day or other you will be sure to have use for them. Iodide of mercury, which is really by far and away the strongest antiseptic we have, is used of the same strength as the perchloride.

Now those I have mentioned are the most commonly used; you will see them advertised in all sorts of powders, soaps, and dressings. An antiseptic at one time very much in use is iodine diluted wlth water to such a strength as to resemble sherry in colour. And then you know very well there is Condy's Fluid, and there are numerous other preparations, such as Izal, Creolin, and Formalin that we need only mention. These I have told you are the most important, and if you remember them it will be quite sufficient for our present purpose. Now do not forget that these lotions used of the strength I have named will kill germs or will prevent them developing, for they will prevent their increasing in number and size.

Besides these lotions we have other agents, such as powders, which are used to sprinkle on the skin. Of course, you all know starch and zinc powders, which you use to dust the skin of babies, but they have no antiseptic value. The chief powder that one uses is iodoform, which, of course, you have all come across as yellow powder with a peculiar smell. Then there is double cyanide, which is most useful and extremely powerful. Boracic acid powder is used extensively and in most cases where a powder is supposed to do good, but I must say it is not very reliable, for one cannot trust to it for killing germs. It is, however, useful for preventing decomposition of meat and for preventing milk from turning sour.

We must now see how these lotions and powders are used, and in order to do this we will just suppose we are going through an operation with any patient. The first thing you do with a patient who is going to be operated upon is that the night before the operation you give him an aperient to open his bowels, because it is no uncommon thing for a patient to have his bowels opened on the operating table, and you can understand that, because of the smell and of the germs that are set free, this gives rise to actual peril to nim. It may be necessary to give a soap enema in the morning to assist the free action of the medicine.

The next thing you do is to see that the patient does not have very much to eat before the operation. If you give a patient a good breakfast and then give him chloroform, he would proceed to immediately bring up his breakfast on the operating table, which might lead to choking and even death before the operation has begun. Children are a little different from adults, and digest their food much quicker; so it is not wise to keep them too long without food, and in the case of babies it is not necessary to keep them long without food at all. As a general principle, however, you must restrict the food of the patient. Even if you have not orders to do so in private practice, you should be very careful what you give a patient who is going to be operated on.

(*To be continued.*)

### Making Things Better.

### A CHAT WITH MISS KATHARINE H. MONK, SISTER-MATRON AT KING'S COLLEGE HOSPITAL.

It is now nearly ten years since Miss Monk commenced to carry out her system, carefully designed to make the nurses more efficient and also to make things better for them. The principle on which in the course of her professional career she has proceeded has been that efficiency in the ward is best promoted by consideration for the workers. It was in accordance with this principle that she anxiously asked herself whether it was possible to put in practice the theory of an eight hours' day for nurses, involving three shifts, but after much deliberation she decided against it as impracticable. But convinced that the long hours were taking the life-blood out of nursing, she finally decided, in 1890, upon a nine hours' day. It was to inquire about the actual results of the working of this valuable reform that I saw her at King's College Hospital.

"One result," said Miss Monk, after she had explained that the system is in force in all wards, whatever their size, "is that we have had much less illness. In fact, unless our nurses get typhoid, they are hardly ever ill. While we used to have six or seven down from tonsilitis and headache, we rarely have one now. Another important result is that instead of sending tired nurses back to the ward we now send them back mentally and physically refreshed, because we give them four complete hours for refreshment."

"Did you," I interrupted, "study the question from the worker's point of view only?"

"No; I tried to look at the situation also from an educational point of view. It is true that you can employ a man or a woman twenty-two hours out of twenty-four; but I maintain that if you attempt to do this the material at your disposal will not produce good work. Perhaps," continued Miss Monk, "it will simplify matters if I mention the three great points I sought to attain."

"By all means. What was the first?"

"I aimed, first, at reducing the hours of work in order that the workers, while obtaining ample rest, should enjoy sufficient off-duty to enable the woman to keep in touch with outside interests, apart from her work—very particularly so with home-life and friends—and thus prevent her from being narrowed or dwarfed intellectually. My contention is that a nurse ought not only to be a power in her work, but also in the world. I want to see her womanhood, as well as her nursehood, raised."

"And, therefore, to keep herself posted up in what is going on outside?"

"Precisely. I consider that it is most useful for her to see a good play, and to hear a good concert; and I took care in arranging the off duty to furnish the necessary opportunities. The nurses here can go home to afternoon tea, or dinner, to a matinée or an evening performance, without interfering with their work. As to my second point," said Miss Monk, "I desired to deal with nurses as pupils. The best Sisters are tempted, when they are going to have a busy afternoon, to keep their sharper pupils on duty, and it is only human nature that they should be. But this would be bad for them, because it is important to maintain the efficiency of the teacher; and it is impossible, under the nine hours' system, because the nurses must go off duty in rotation, which means that the Sister may often find herself landed, on a busy afternoon, with the dullest or the least efficient pupil. The Sister has therefore to bring her best powers to the front, and the pupil to show what she is made of."

"Can the Sister change the time?"

"Not without my authority. You see the great value of the arrangement is that it affords the Sister a better chance of forming a true estimate of the pupil's capacity; and last, but not least, while putting the dull girl on her mettle, it prevents the sharp girl from being spoilt because she sees that she is not picked out for special work."

"How does the system affect the whole standard of work?"

"That is what I am coming to. The third point I had in view was to raise the whole standard of the work, morally and technically; to put it on a fairer and sounder basis; to obviate the difficulty of a third shift; to encourage the workers, when on duty, to work with greater energy; and to give them the grand opportunity of keeping in touch with the outside influence, which is often elevating and most wholesome."

"And how far has this been accomplished?"

"There is every reason to be satisfied. You understand, of course, that the work always stands first. Patients appreciate a woman of culture who is in sympathy with outside movements, and that is why, instead of favouring dramatic or musical clubs in the hospital, I only allow a newspaper club. Other clubs tend to narrow their interests. I want them to forget that they are nurses when they are outside. Contact with the world makes them more womanly, and there is no profession in which women should be more womanly than in that of nursing."

"It would be interesting if you could tell me what the order of things was when you were a nurse."

"We used to work from seven in the morning until ten at night. Our hours off duty were two hours a week—from six to eight. We returned to the wards on those evenings at eight, and worked until ten p.m. It was impossible for us, in those circumstances, to take an interest in any outside movement, and many splendid women I knew were quite spoilt by the narrow life which was unavoidable."

"How about the material?"

"As well as the long hours, we had less than half the material of present days. In a ward of thirty beds we had a sister, a staff-nurse, and a probationer. Now, we have in the same ward a sister, a staff-nurse, and four probationers. Then there was only one night nurse; now, there are two and a night sister always to refer to. In fact, the conditions are quite different."

"What are the present hours of night nurses?"

"Their hours practically stand at eleven. They go on duty at nine p.m. and leave at nine a.m., but I oblige them twice during the night to go into the day-room for half-an-hour for a comfortable meal. They are only on night duty for three months, and in that period they are given monthly two days and two nights out of the hospital. When I made a calculation for the year there was little difference. Their lot is not so hard as it looks."

Miss Monk then proceeded to emphasise the importance of nurses being prevented from thinking that their lot is hard. As she observed, "The tendency in the present day is to make the life of a nurse too mechanical. Nothing that will have the effect of militating against the true spirit of nursing ought to be encouraged. One notices a disposition on the part of some women to make too much of themselves. Nurses, while of course requiring to earn enough to provide themselves with bread and butter, imperatively need the sacred love of human life as the governing motive of all their actions. They must be prepared for self-sacrifice, and willing to give up a part of their life."

"What holidays do the probationers get?"

"The probationers have one whole day a month, and three weeks in the year. The day staff nurses have from half-past four on Saturday afternoons until ten o'clock on Monday morning once a month, and four weeks in the year. In each case two extra days are allowed for travelling. Moreover, we never hesitate to send nurses away for a few days if they are a little run down, and this is not taken from their holidays."

"What happens if a nurse is more seriously ill?"

"We have a reserve staff, which is used for taking the place of sick nurses, for doing special duty when special nurses are required for any special cases, for taking the work of the night nurses when they are off duty, and that of the probationers when they are off day duty."

"Of what is the reserve staff composed?"

"The bulk are probationers, but a few are staff nurses to take the place of staff nurses who for any reason are off duty. They are not always on the reserve staff, but they continually change, and very frequently before they have been on reserve duty a month they go on regular duty."

"What is your method of testing an applicant's suitability for the work?"

"Every nurse is tried for one month, and if at the end of that time the report of the ward sister is satisfactory the probationer enters into a contract for her three years' training. During the month of trial I do not care whether she can make a poultice or not; but I want to know whether she is punctual, quiet, neat, clean, active, reliable, careful, obedient, patient, good-tempered, observant, and intelligent. She is put into the wards to learn all she can, but little is expected of her during the month. I ought to add that the nine hour system is very merciful to probationers. The first six months are very trying to them, and under our system they always secure once, and sometimes twice a week, a long night. That is to say, when their off-duty time falls between seven a.m. and eleven a.m., they are allowed to breakfast at half-past nine instead of half-past six. This is a privilege which shows, in a striking manner, how the training of nurses has been ameliorated without losing sight of the main object in view."

### The Home for Confirmed Invalids at Highbury.

Opening by the Duchess of Albany.

On Wednesday the Duchess of Albany visited the new house recently acquired by the council of the Home for Confirmed Invalids, Aubert Park, at 1, Highbury Terrace.

Her Royal Highness, who looked remarkably well, was accompanied by the Hon. Mrs. Morton, and arrived punctually. She was attired in a dress of dark grey silk, relieved by black velvet collar and cuffs trimmed with heavy white lace; and wore a bonnet of black and yellow straw.

The Rev. Prebendary Barlow, D.D., vicar of Islington, received and conducted her to the tea-room, where the members of the Reception Committee were introduced. Proceeding to the reception-room she was presented with a bouquet by Miss Silvia McLaren.

The Rev. C. H. Banning, M.A., offered a short prayer, and Mr. David Howard, J.P., thanked Her Royal Highness for her visit, and shortly explained the object of the homes. Referring to the old home in Aubert Park, he said that to many desolate sick ladies it had been a "Land of Beulah," and that the new home would be a stepping-stone to happiness for many more.

Then the Duchess, rising, said in a clear pleasant voice, "I pronounce this Home open." Resuming her seat, she received a number of purses from a band of attractive children and young ladies, the amount realising from £80 to £90. The "patients' purse" was presented by the little daughters of the hon. physicians, Drs. Kelly and Stokes, to whose kindness all connected with the home owe so much.

The Duchess next visited the charming stalls arranged in the various rooms, and made several purchases, winning golden opinions by her gracious manner.

After tea, Her Royal Highness left for the old home in Aubert Park, where Mrs. Baker, of Ythandale, Wimbledon, a lady who has been connected with this charity for over half a century, received her.

The new home is an old house admirably situated at the end of Highbury Terrace, overlooking the park on two sides. The rooms are cheerful and very comfortable. There are six bed-rooms, a dining-room, sitting-room, and a small sitting-room upstairs for those who cannot easily go up and down stairs. There is accommodation for thirteen ladies; and in a couple of years the lease of the adjoining premises will fall in, and the council will be able to increase that number to thirty—the same number as at the Home in Aubert Park. Last year there were 120 applications for admission.

### Practical Hygiene Teaching in Elementary Schools.

Miss Alice Ravenhill read an interesting paper on "Practical Hygiene Teaching in Elementary Schools" at the last sessional meeting of the Sanitary Institute. Beginning by the statement of the deplorable fact that at present not more than 1 per cent. of the five and a half million children whose names appear on school registers receive instruction in any branch of sanitation, whether included under the head of domestic economy or specifically as hygiene, Miss Ravenhill pointed out how the various objections to the early teaching of hygiene to both boys and girls could satisfactorily be met. She laid especial stress upon the economic value of widespread preventive knowledge in health matters, the importance of exciting intolerance of insanitary conditions in quite early life, and the need for awakening zeal in the teachers for this subject, which courses of lectures in the training colleges would help to achieve. "It is to education," continued Miss Ravenhill, "that we must look to bring home to the people that it rests mainly with themselves to work out their own salvation from sickness, poverty, and sorrow, by letting in the light and air of hygiene into the dark places of ignorance, apathy, and prejudice. Can such education begin too early in life? The instruction is of equal importance to both sexes, and throughout the country. Many miserable hovels in so-called healthy villages are as bad in respect to overcrowding and dirt, want of thrift, and dense ignorance, wasted food and sickly occupants, as any city slum, and it is against these and other evils that the rising generation must be armed if our great population is to have a fair chance in the race for prosperity." Miss Ravenhill then described in outline her practical scheme for the wider diffusion of hygienic knowledge in elementary schools, and instanced the success which had attended the introduction of the subject into the time-tables of the Leicester schools, through the zeal of Mr. Major, inspector under the Leicester School Board. She gave an example of a lesson, showing how the points should be developed, and illustrated by models, objects, and pictures, dwelling on the fact that the treatment in detail must depend upon the zeal and conscientiousness of the teacher, to whom a free hand and full scope should be allowed for application to local conditions. Briefly, the chief points contended for were these—the early teaching of simple hygiene to both boys and girls in elementary schools, and the inclusion of this subject in the curriculum of the training colleges. Miss Ravenhill made it clear that hygiene and sanitation might be thus added to the school time-tables without in any way increasing the educational burdens of either teacher or taught.

In the discussion which followed much approval of Miss Ravenhill's scheme was expressed, and the speakers included Dr. Gladstone, Dr. Kenwood, Professor Notter, Mr. Macan, Dr. Kimmins, Dr. Warner, Dr. Shuttleworth, Mr. Major, and the Rev. T. W. Sharpe, C.B., chairman of the meeting.

### Metropolitan Nursing Association.

The annual meeting of the Metropolitan Nursing Association was held at Grosvenor House on Friday. The chair was taken by Mr. Bonham-Carter, in the absence of the Duke of Westminster, who was prevented attending by indisposition.

The report shows satisfactory progress. The complete staff of the association now consists of a superintendent, a senior and two staff nurses; and twenty-five district nurse probationers were trained for Queen Victoria's Jubilee Nurses' Institute. Lectures provided at the expense of the institute were given at the Central Home on "Diseases of Women" by Mrs. Scharlieb, M.D., and on "Hygiene" by Mrs. Goslett, member of the Sanitary Institute; Miss Earle also gave two courses of demonstrations on cookery. The Duke of Bedford kindly allowed the committee of management an extra year upon the termination of the lease of No. 23, Bloomsbury Square, in order that they might weigh the question of renewing their tenancy upon proposed terms, or of removing to another locality; but it is stated that many circumstances bearing upon the matter require consideration before a decision can be arrived at. The total number of cases attended during the year, including 1,155 Board School children, reached 2,157. A nurse also attends Bloomsbury Dispensary for an hour daily. The health of the nurses has been good, and numerous grateful letters of thanks have been received from patients for the work of Miss Gray and her staff. The chief items in an income of £1,523, were subscriptions amounting to £687; and payments for training nurses from Queen Victoria's Jubilee Institute, £627.

The Chairman moved the adoption of the report, and Mr. Ernest Flower, M.P., seconded the motion. Mr. Flower, in his remarks, dwelt chiefly upon the importance of the lectures provided by the Queen's Institute, because, he said, the advance of knowledge in so many departments made it essential to keep the nurses fully instructed; and upon the value of the work of the nurses at the Board schools. He feared that the medals offered for the children's regular attendance in some degree encouraged their return to school sooner than was desirable after infectious diseases. The report was accepted unanimously.

Mr. Bousfield proposed the second resolution pledging the meeting to support the association. He remarked that the work of the district nurse must play an important part in the problem of rearranging the hospital charities of the metropolis. He congratulated the association upon the small contribution they had so far received from the working men themselves, and trusted that in the future the nurses' associations would be affiliated with clubs, although an attempt some time ago to bring this about had failed.

Dr. Murray Leslie seconded the motion, and gave some interesting details of the nurses' actual work in the East End.

Mr. Harold Boulter, hon. secretary Queen Victoria's Jubilee Institute, supported it, and read an extract from one of the Institute's inspector's report in a district not three miles from Grosvenor House. He said the work of the Association was not so obvious to the rich as it was useful to the poor, but in two palaces a deep interest was always taken in it, namely, Windsor Castle, where the Queen herself occasionally received her nurses; and Grosvenor House. He could not help thinking how easily £100,000 had been raised for a college at Khartoum, and how, not so long ago in that very room, a large sum had been collected for the distressed Armenians, whilst the Queen's Institute wanted £10,000 to spend, and could only obtain £5,000. He added that it mattered little whether the public supported the Queen's Institute or the Metropolitan Nursing Association, for the two associations were so closely allied that what benefited the one helped the other also.

Mr. Mocatta, in proposing the re-election of the committee, said how sorry the Duke of Westminster was at his enforced absence, and the Rev. Dacre Craven proposed a vote of thanks to his Grace for lending Grosvenor House for the meeting.

### Where to Go.

The New Gallery.—This charming gallery contains a very interesting and representative collection of English contemporary painting this year. Shannon, Alma Tadema, Alfred East, Edward Fabey, MacWhirter, W. B. Richmond, Sargent, Wilfred Ball, Sir H. Clay, John Collier, Watts, and numbers of others show excellent representations of their powers. Mr. Watts' portrait of Lord Roberts and Mr. Holman Hunt's "The Miracle of the Sacred Fire" have excited a great deal of attention. The last picture is a brilliantly-coloured scene quite crowded with life—so much so that it requires close attention to discover the full meaning of the picture. The catalogue gives a full and graphic account, with the help of which the story depicted becomes clear. Mr. Watts shows two other pictures besides his portrait of Lord Roberts, one of which aims an arrow against the fashion which ruthlessly sacrifices the brilliant plumaged birds to its dictates. Mr. Shannon's portraits cannot fail to charm, they are so delightfully graceful. Mr. Sargent, to whom the visitor is apt to look for some of the most striking and fascinating pictures exhibited there, shows only one portrait this year. It is a fine piece of work, quite as worthy of his talents as the more sensational canvasses he usually exhibits. There are some very good landscapes, but they show no great originality of treatment or subject.

The Fine Arts Society.—Here are some brilliant examples of colour in modern art by Gaston La Touche, well worth a visit.

Dowdswell Gallery, 60, Bond Street.—The interesting collection of original drawings illustrating the Rubaiyat of Omar Khayyam, by Elihu Vedder, are attracting a number of visitors.

The Guildhall.—The splendid exhibition of Turner's works shown at the Guildhall should not be missed. Such an opportunity may not return for a very long time.

The Working Ladies' Guild.—A summer sale in connection with the above guild will be held at Windsor House, Mount Street. It will be opened on Friday, May 5th, at three p.m., by H.R.H. the Princess Henry of Battenberg, and will continue open from two to six on May 5th, and from twelve to six on May 6th.

The New Building for the Hampstead Hospital.—Garden Fête by the Ladies' Association of the Hampstead Hospital in the house and grounds of Golder's Hill on June 1st, at three p.m., in aid of the Building Fund. The entertainment is under the patronage of H.R.H. the Princess Christian of Schleswig-Holstein. Tickets may be obtained from the Hon. Secretary of the association, Parliament Hill, N.W.

Messrs. Shepherd's Spring Exhibition.—At 27, King Street, St. James's Square, Messrs. Shepherd are showing an interesting collection of early masters. Amongst the portraits is one of Dr. Mead, physician to Queen Anne and George II., and to St. Thomas's and St. Bartholomew's hospitals.

### To Nurses.

In order to increase and vary the interest in the *Mirror*, we invite contributions from any of our readers in the form of either a paragraph, a letter, or information, and will pay a minimum of 5s. for each contribution.

### Echoes from the Outside World.

If you are not too tired to enjoy it, run in to the New Gallery the next time you get an hour off duty. You will have no doubt as to the picture which is attracting the most attention, for there is always a little crowd round Mr. Holman Hunt's "Miracle of the Sacred Fire in the Church of the Sepulchre at Jerusalem." If you will take my advice, sit down first of all and read the two and a half pages of letter-press about it which you will find in the catalogue. It is impossible to understand the picture without doing so, and it is impossible to enjoy the picture without understanding it. You will be most impressed with the marvellous and bewildering mass of colour. The criticisms will amuse you. I overheard one man remark, "It is the grand work of a grand master," whilst just behind me a woman was declaiming with scorn, "That is not art; it is only the reproduction of a motley crowd." But the exclamation of a young girl who had only just approached the canvas seemed to me quaintly *apropos*. She gazed for a second, and then said plaintively, "Oh, dear! I wish I knew which corner to look at first!" I had just the same sort of feeling myself. You will find another little crowd round Miss Alma Tadema's "The Closing Door." She has this year scored a bigger success than either her father or her mother, and, moreover, sold her picture at once for £300. The picture of a war correspondent in white flannel also appeals to quite a number of people because of the magic name of the Sirdar. It is called "With Kitchener to Khartoum," and is a portrait of the author of the book. Mr. Watts has made an appeal to all women who wear feathers in their hats. He has portrayed a majestic, blue-robed figure, her face buried in her hands, bending over an altar upon which are a mass of slaughtered birds. He has called the work "Dedication," but the reason why is not very clear to me. Don't miss Mr. Alfred East's "The Land that Shakespeare loved," nor the full-length portrait of Lady Henry Bentinck, by Mr. Shannon, "all in a garden fair." It is difficult to know which to admire more, the lady or the hollyhocks.

I wonder what has imbued English men and women in the closing years of the century with such a strange craze for commemorating all sorts of anniversaries? There was a time when commemorations were limited almost entirely to St. Valentine's and Guy Fawkes' days, but things have changed since then, and if the mania goes on increasing at its present rate the most conspicuous days in the year will soon be those to which no particular significance is attached. Already the dates when King Charles the Martyr and Lord Beaconsfield died, and when Lord Nelson gained the battle of Trafalgar, are marked by the offering of floral wreaths, by meetings and demonstrations; the admirers of Mr. Gladstone talk of wearing white roses on the anniversary of his death on May 19th; the Millenary of King Alfred the Great with its prospective jubilee is looming ahead; and during the present week there have been the Cromwellian Tercentenary, the Shakespeare Celebration, and an increased recognition of St. George's Day, which fell on Sunday. Flags with St. George's Cross upon them were displayed on many churches, and some of the people in the streets wore red or white rosebuds. A few of the ladies in Hyde Park who were present at what is grotesquely called "Church Parade," after real, or supposed, attendance at morning service, appeared with bouquets and buttonholes of our national flower. The persons who rejoice most over this recent development are the flower-growers and the flower-sellers, for to them it means a golden harvest.

Here are two items of dress news, straight from the world of Fashion, which I think will amuse you. Tailors and dress-makers are getting seriously concerned about pockets. The new "eel-tight" dresses are simply spoilt, they say, if a pocket of any kind is allowed. At first obliging costumiers tried to humour their "clients," and inserted one in the back width of the skirt, hidden up somewhere beneath five or six buttons and loops, all of which had to be undone before the handkerchief could be procured. Now even this luxury is no longer permissible, and those ladies who are not heroic enough to dispense altogether with such an old-fashioned article have a small one inserted in the *hem* of their gowns. Thieves will at once have to start practising how to pick such strange pockets, for their well-known tricks will be unavailing. The other item relates to sleeves. The most fashionable come down as far as the knuckles, so that only a scrap of hand can be seen to peep out. But experience has taught the wearers that it is difficult to keep the bottom of the sleeves from rolling back, and as it is most undesirable, apparently, to show anything but the tips of the fingers, a loop of ribbon is sewn on and slipped over the thumb. This keeps the sleeves from "riding" up, as it is called. Because I tell you of these two absurd innovations, please don't believe that I imagine for a moment you are likely to introduce them to your new frocks, for holiday wear. It is impossible to fancy a nurse devoid of common sense, and no one with even a ha'porth of this useful commodity would put her pocket on a line with her ankles!

It is rather the custom to run down everything "made in Germany," even while we gladly use the articles, which we obtain at a cheaper rate because they are not of home manufacture. But one society which has just started in Berlin seems to me so admirable that I must tell you about it, even at the risk of being considered unpatriotic. The name of the new organisation is "The Society in Aid of Poor Actresses," and the members who belong to it pledge themselves to help in every way they can the struggling, humbler actress, who is paid a very small salary and yet has to appear well dressed upon the stage if she would keep her engagement. No theatre in Germany, except the royal ones, supplies any dresses, so that frequently it is only by going into debt largely that the average actress can get along. To avoid this the committee of the new society appeal to the ladies of the aristocracy to send to them such "cast off" dresses, mantles, hats, gloves, &c., as they may no longer have a use for, so that they may be distributed among the needy. There exists in England a society on somewhat the same lines, but its design, I believe, is principally to help those who are sick or out of employment, though naturally sometimes the question of dress comes within the range of its charitable deeds. All our principal actors and actresses help so generously that the aid of the outside public is seldom required. In this, apparently, the Berlin society differs from ours.

I don't know whether you sometimes get as tired of the regular routine of beef and mutton as I do, but if so, you may like to know that an attempt is to be made to introduce bear's flesh into England as an article of food. The Parisians—who are much bolder in trying new dishes than we are—have already pronounced it excellent, so perhaps we shall think the same when we get a chance of trying it. The bears will arrive, of course, in a frozen condition, and will mostly come from Siberia. Moreover, the Californians contemplate sending us a new fruit, called the coral berry. It grows on a bush inside a burr, which gradually opens and exposes its contents. It is slightly acidulated, and has a delicious scent. Those who know it best say that it is almost as good as the strawberry. If this is true it is sure of an English welcome.

### Across the Seas.

### SOUTH AFRICAN HOSPITALS.

### III.—The Question of Dress.

A few Boer women have recently begun to train as nurses, but the Dutch colonials do not take very kindly to hospital life. At all the hospitals under Boer control the nurses are very hard worked, the hours long, and the general curriculum more laborious and trying than English nurses have any conception of. The food in Boer-governed hospitals, as in the colonial hospitals generally, is extremely good. But Dutchmen find it hard to realise that white women cannot do as much as Kaffirs—not that Kaffirs work hard, they are only expected to do so. In many of the Boer hospitals the staff is constantly changing on account of hard work. Nurses come on a month's trial and give a month's notice. This is especially true of Englishwomen, who certainly do not show the same capacity for sheer hard work as the colonials, a result probably due to the fact of working in a climate to which they are not accustomed. The colony is undoubtedly healthy, but very different from that of England. Personal observation leads me to the conclusion that the climate affects the working capacity of the English nurse more than it affects her health. It produces a certain sense of languor which is apt to cause the amount of work expected from a nurse in an average English hospital to appear somewhat formidable.

Wherever our colonial hospitals fall into Government hands or are under Government inspection the result is good. More harmony and a more common-sense uniformity of system is observed in the Government hospitals. This is natural enough since the Government system is based more on a pattern which suits colonial conditions. It is formulated through trial and experiment, and is the sum of the combined experience of colonial experts, who naturally are better able than newcomers to frame rules which fit in with the social and industrial conditions of the colony. All colonials who know anything of hospital matters feel a great indebtedness to the mother institutions of England for the standard the latter have set and the perfection of their methods. At the same time they cannot help recognising that British systems need specialising to meet colonial needs. In so small a matter as the question of uniform, colonial nurses have had to suffer various tribulations through the attempts made by English matrons to fit the clothing suitable to London to the climates of South Africa—a feat which cannot be achieved. At one hospital the English matron issued the strictest rules with regard to the wearing at all times by her staff of a London-designed out-door uniform. No nurse was even to be seen on the steps of the hospital in anything but this long-cloaked, black bonneted uniform. Now, the climate of South Africa reduced such a regulation as this to an absurdity, especially as strict injunctions were given that nurses going to garden or tennis parties should wear their out-door uniform and play tennis in hospital bonnets. In a short time the staff was in revolt, as it might well be, at having to play tennis in a semi-tropical climate in an unshaded Bart's bonnet. The nurses resolutely stayed indoors, the committee held an inquiry, and abolished the out-door uniform—an uncomfortable fact for the matron. If she had tactfully devised a cool, light, and pretty outdoor dress, and gradually educated the colonial nurse to the point of professional evolution of recognising that out-door uniform has many commendable qualities, all would have been well; but suddenly to put a veto on the light, cool muslins and shady hats the staff had been accustomed to wear in off-duty times, and to substitute for these the black bonnets and cloaks suited to a London climate, was to court absolute failure. Another matron abolished, by the issuing of a decree, the wearing of white shoes by her nurses. But all colonial women wear white shoes. They are cool, and suited to a hot and dusty climate. The matron said it was not professional, because English nurses do not wear white shoes. But that was not the point which chiefly interested colonials, who do not recognise that all their customs must be "made in London." The girls who had worn white shoes since their first infant bootikins declined to substitute heavy thick black ones in order to suit London ideas. There was a tremendous tug of war and an incredible amount of friction over so simple a matter as the wearing of the white shoes. The matron had to revoke her edict, which was manifestly a mistake, because it took no heed of established customs and the exigencies of the climate. It would be easy to go on adding to the list of failures resulting from attempts to reproduce here, thousands of miles away, in faultless miniature, the internal regulations of English institutions.

But I will pass from such trifles as bonnets and shoes—though weighty bonnets and heavy shoes are no trifles in the heat of some of our summers—to wider social considerations whereon English and South African ways can never be made to meet. From what I have already said, I feel sure my readers will have come to the conclusion that it would certainly be wise for newcomers to the colony, whether they be private nurses or nurses appointed to institutional positions, to study our accepted social customs and established ideas before introducing reform, and attempting to alter in a week the views on such matters to which the colonial has been born and bred. Our social customs differ widely from those in the "Old Country," the difference being, perhaps, as striking as exists between the customs of England and America. The hard and fast regulations which are accepted in England as being right and proper for hospital discipline do not, and cannot, commend themselves to colonial women at the present time, especially since the staffs of South African hospitals are not drawn from the same social class as at home. It must be remembered that we are young and new, and our very youth prevents us from regarding social conditions from the English standpoint. Neither have we the same educational advantages which are enjoyed by our English sister nurses. To sum up, we must be permitted to exercise our right of ordering our own society as we please, and as seems suitable to the colony, for we are in no way bound to accept without question the infallibility of the British standpoint. Loyal as we are to the Mother Country, we do not attempt to criticise or to compare English ways with ours to the disadvantage of the former, for we thoroughly grasp the principle that manners and customs depend a great deal upon climate.

### Presentations.

Miss Minnie Love, "Queen's Nurse," Newtownards, Ireland, has recently been appointed Head Nurse and Superintendent of Ballymena District Nurses' Home, and on leaving Newtownards was presented by her committee with a handsome dressing-case, silver mounted, accompanied by sincere wishes for her future success.

### Resignations.

Miss E. M. Pringle, matron of Basford Isolation Hospital, Nottingham, has resigned her appointment after three years' service. It is with much regret the hospital committee have accepted her resignation, and whilst congratulating her they wished her every happiness in the new life which she is about to enter.

### Our American Letter.

The life of a woman who has divorced her husband is by no means easy even in America, where the marriage contract is so readily annulled. A lady of irreproachable character divorced her husband for non-support, cruelty, and drunkenness, and being fond of nursing managed to obtain a short training in a small hospital. She had, however, first been refused admittance to several private homes and institutions because of the divorce she had obtained. Here, too, after six months' training she was requested to resign her position in the school which had accepted her for the same reason. Her capacity for the work, however, procured her the countenance of the doctors, and she would have succeeded very well but for the fact that her husband continually pestered her to re-marry him. At last she consented, and shortly afterwards, having nursed him faithfully through the last stages of consumption, the result of hard drinking, she became a widow. The sequel to her story is the strangest part of it. She applied for readmission to the training school, and as she was now a widow it was graciously conceded. There must be something wrong in a system that makes it impossible for a good woman, because she is divorced, to secure the means of earning a livelihood.

Many of the New York nursing institutions are very inadequately supplied with sleeping accommodation for the nurses. It is reported that in one large institution it is impossible to get a separate bedroom, even small rooms being fitted up with two beds and sometimes used by two more nurses as well as their legitimate occupants for a dressing room. Part of this home was called the "stalls," and each stall contained two beds and only one small wash-hand basin. In another institution seven nurses occupied two small rooms, and at an obstetrical hospital the nurses had to make use of the operating room for their toilettes. The writer of the article from which these statements are taken complains of "want of comfort," but it might be asked what the sanitary authorities are about to allow such disgraceful overcrowding.

The number of nurses trained at the Bellevue Training School has reached the large total of 580. Of these 67 are holding posts of matrons, head nurses, or superintendents, two are in China, two in Turkey, and one in India as missionaries, 108 are married, and nine have studied medicine, a very satisfactory record as far as can be judged by numbers alone.

Mr. Harris C. Fahnestock has decided to build a new training school for nurses in connection with the New York Post-Graduate Hospital in memory of his wife. It is estimated that the cost will be £20,000. It will be equipped as perfectly as possible, and will be known as the "Margaret Fahnestock" Training School.

The chief lady superintendent and the superintendent nurses of the branches of the Victorian Order of Nurses, Canada, are graduates of the Waltham Training School, Boston. This school is cramped as regards quarters, and efforts are being made to collect money to provide suitable buildings for it. A large and suitable site has been obtained, and a handsome building is in process of erection. £8,000 will be required to defray the cost, but half that amount has already been subscribed.

A home, providing accommodation for seventy-six nurses, will be built shortly in connection with the Presbyterian Hospital, Philadelphia, Pa., by Mr. J. Renwick Hogg, in memory of his father. Mr. Hogg is one of the trustees of the hospital, and the site on which the new home will be built is hospital property.

A training school for nurses has been started at the Craig Colony for Epileptics, New York. The probationers are especially taught cooking, as diet is of great importance in nursing this disease.

### Examination Questions for Nurses.

Nurse Menzies-Jackson is the winner of the first prize, and Nurse H. L. Arnold of the second. Many excellent answers have been sent to this practical question. The above-mentioned ladies are the successful competitors, because they recognise the necessity of filling the space under the grate with something that shall obviate any noise from falling cinders. Nurse Menzies-Jackson also remembers the tendency of paper bags to crackle, and recommends muslin for the purpose. I would again recommend candidates to be more careful of their writing, spelling, and the general neatness of their communication. Carelessness in such matters argues a slovenly habit. Take notice that the competition *closes* a fortnight from the date of the publication of the question, and that all letters must have "Examination Question" written on the outside of the envelope.

First Prize.

1. Select carefully fairly large lumps of coal to supply your scuttle. These may be wrapped in soft rag, muslin, or thin paper, that will not crackle. Place the knobs upon the fire with the hand, protected by a loose glove or bag made of some soft material. Under the grate, and well around in front of it, spread a carpeting of moss, or a thick layer of sand, to enable the cinders to fall noiselessly.

2. In order to arrange a fire to last all night, the cinders should first be carefully raked away from the back, sides, and front of the grate; the spaces thus left should then be filled in with small lumps of coal, also the spaces between the bars, but not too closely. Next select some medium-sized cinders and spread them upon the fresh coal, again another layer of coal in small lumps, and then bank up the larger cinders on the top; towards the chimney, but not too high; a small handful of salt or sand may then be scattered over all. The top layer of cinders may be moistened or dipped in water before placing them.

Second Prize.

To make up and keep going noiselessly a fire, I would put a thick layer of sand or fine ashes in the fender, wrap the coals in small paper parcels, and put them on with my fingers; use a stick for a poker.

To make up a fire to last all night without attention, I would mix cinders and small coal, damp the mixture, draw the fire forward, and fill up the back tight with the mixture; or use a briquette.

Question for May.

State what steps you should take to prevent the spread of infectious disease, such as scarlet fever or small-pox, if called to nurse such a case in a private house.

### Minor Appointments.

Keighley and Bingley Joint Isolation Hospital Board.—Miss Helena M. Morton was appointed Charge Nurse on April 15th. She was trained at the Richmond, Whitworth, and Hardwicke Hospitals, Dublin, and for four years was charge nurse at the Borough Fever Hospital, Southport.

Forster Green Hospital for Consumption, Belfast.—Miss Ellen L. Little has been appointed Sister. She was trained and for two years sister at the Richmond, Whitworth, and Hardwicke Hospitals, Dublin.

Falmouth Cottage Hospital.—The new Matron here is Miss Rosie E. Merrett, who was trained at Guy's Hospital, London; and afterwards assistant matron at St. Mary's Hospital, Manchester.

### Everybody's Opinion.

[Correspondence on all subjects is invited, but we cannot in any way be responsible for the opinions expressed by our correspondents. No communication can be entertained if the name and address of the correspondent is not given, as a guarantee of good faith but not necessarily for publication, or unless one side of the paper only is written on.]

### THE CLOTHING OF PROBATIONERS.

"MATRON" writes: Circumstances having directed my attention to the clothing worn by hospital nurses, I think it would be wise for matrons to impress upon probationers the importance of warm and suitable underclothing. I believe this to be one cause of nurses' health failing and of the readiness with which they fall victims to influenza. I have been surprised to find nurses wearing the thinnest of calico, shoes and stockings fit for the drawing-room, dainty, perhaps, but lacking wisdom. If they become ill the work is blamed, not the absence of clothing suitable for variations of temperature in wards and corridors.

### A REMINISCENCE OF THE NORTH-EASTERN HOSPITAL.

"AN OLD NURSE" writes: I see in your columns a most interesting account of the work going on under the Metropolitan Asylums Board hospitals. It brought back to mind the time I joined the North-Eastern staff as a charge nurse. Being a fully certificated and trained nurse of some years' standing I learnt a very great deal I had not had the chance of learning in other hospitals and infirmaries to which I had belonged. The morning after my arrival I entered the wards at seven a.m., when I was informed that a nasal feed was due at eight a.m. I had never seen one given, or even prepared one, so I instantly said, "I cannot give it, I do not know how." It was a source of intense amusement to one of my first assistant nurses, who laughingly told the doctor that they had "a rum sort of charge" who did not know much about nursing. The doctor's reply was, "Nurse, I will teach you how to give it, and I admire you for the candid way you owned that you could not undertake it, instead of trying and hurting your patient." I learnt a lesson, and I think those nurses who stood by whilst the doctor taught me learnt one also, namely, never to be too proud to own what one does not know.

### ARE NURSES EXTRAVAGANT?

"A NURSE OF TWELVE YEARS' EXPERIENCE' writes: As a rule, certainly yes, more especially private nurses who are on the "co.-op." principle. These, as a rule, on leaving a case, have £6 or more in their purse, and they immediately have just whatever pleasures they fancy—dinners at the "Holborn," a visit to the theatre afterwards, and a hansom there and back. Most private nurses get used to living in an extravagant manner at their cases, and, sad to relate, they rarely think of the future. One hears nurses saying, "Oh, when I have finished this case I shall go down to Brighton; I always have a 'good time' between my cases." This means £5, or so. I am no advocate for nurses not enjoying themselves, but I think a meat tea at an A. B. C., and pit seats at a theatre ought to satisfy our wishes. I can hear nurses calling me a mean old frump, but let me tell them I have often been and enjoyed myself, and then felt I could still hold some funds for the future. Hospital nurses, from their regular disciplined lives, are rarely very extravagant, but private nurses are very careless. They have money and spend it freely—2s. 6d. for their tea and a cab back is very usual. To nurses as a body I think the co-operative principle has had a deteriorating effect; they have become more grasping and extravagant, less self-denying and careful, and I often wonder how many individual nurses are better off at the end of 10 years' work than they used to be 10 years ago. I fear we are all very pleasure-seeking, but as long as our first thoughts are always for our patients, we may, and shall, enjoy our pleasures small or great. But we ought to be business-like, and remember that we neither hope nor want to work when we are old and worn, and there are few nurses at 45 who will feel that they can do as much as they could 15 years before; and if they spend what will they do? I have heard, to me, dreadful rumours of the way nurses bet on races, which I also suppose is the outcome of having cash in hand. Surely this is wicked, reckless extravagance, and yet I fear it is true.

### Appointments.

HAVIL STREET INFIRMARY, CAMBERWELL.—The Guardians of Camberwell have arranged with the Local Government Board that the Havil Street Infirmary shall for the future be a recognised training school for nurses, and Miss Frances E. Marquardt has been appointed as the first Matron. After having been trained at the Royal Free Hospital, of which she was subsequently sister, Miss Marquardt accepted the post of superintendent nurse at the Barbados General Hospital. The climate, however, proved too trying and she resigned. She then became night superintendent at Greenwich Infirmary, an appointment she retained for five and a half years. She next became superintendent nurse at Birkenhead Infirmary, and here she organised a recognised training school for nurses. Her experience in this work will prove valuable at Camberwell, where suitable candidates for training will now be accepted.

GUILDFORD MISSION WORKHOUSE INFIRMARY.—On the 22nd inst. Miss Annie J. Watson was appointed Superintendent. Her previous appointments have been superintendent of St. George's Hospital, London; and superintendent and midwife at Christ Church Union, Stepney. On the 8th inst. Miss A. M. Landsborgh and Miss J. E. A. Jones were appointed Charge Nurses. Miss Landsborgh was previously at the Holborn Union and the Golden Road Workhouse, Peckham; and Miss Jones at the Whitechapel Union.

MIRFIELD AND LIVERSEDGE JOINT FEVER HOSPITAL.—On the 7th inst. Miss Ada Williams was appointed Matron. She was trained for two years at the Monsall Fever Hospital, and for three years at the Preston Infirmary. Miss Williams has been sister at the Borough Fever Hospital, Hull; and charge nurse at the Brook Fever Hospital, Shooter's Hill.

ESTON URBAN DISTRICT SANATORIUM.—On April 19th Miss Lavinia Morgan was appointed Matron. She was trained, and afterwards charge, nurse for two years at the Royal Infirmary, Newcastle-on-Tyne. Miss Morgan has since been engaged in private nursing, and for the last fifteen months has been charge nurse at Eston Hospital.

QUEEN CHARLOTTE'S HOSPITAL.—Miss Emily Barry, assistant-matron of the Hospital for Consumption and Diseases of the Chest, Victoria Park, has been appointed Superintendent of the new Nurses' Home.

### Women's Total Abstinence Union.

AT the Temperance Hospital last week Miss Lucas was "At Home" to the members of the Deaconesses' and Nurses' National Total Abstinence Leagues, many of whom availed themselves of the opportunity to visit this beautiful hospital, which the Hon. Mrs. Eliot Yorke referred to as the "best temperance speech one can have." Solos from Miss Beaver, Miss A. Beaver, and Miss Paget added much to the enjoyment of the meeting, which was entirely of a social character. The guests were conducted over the hospital by Alderman Vezey Strong, Captain Sheffield, and Miss Lucas. A vote of thanks to Miss Lucas and the Hospital Board was moved by the Hon. Mrs. Eliot Yorke, president of the Nurses' N.T.A. League, and supported by Lady Elizabeth Biddulph.

### Travel Notes.

By Our Travelling Correspondent.

### XX.—EXCURSIONS ROUND MENTONE.

### Those Suitable for Invalids.

Enthusiasts on the subject of Mentone declare that there are more practicable excursions round that favoured spot than anywhere else along the coast, but I think that must be set down rather to partiality. Still it is quite true that the environs of Mentone are strikingly beautiful, and in spite of the mountainous character of the country there are many excursions which can be made the whole way by carriage, and are therefore suitable for invalids. Among these we may place first

### Gorbio.

This is a drive of five miles each way, and through delightful scenery. The little town itself resembles the other fortress-like towns; there are the same arched and tunnelled footways, the same cruel cobblestones—a terror to the tender-footed pilgrim—the same quaint and silent little church dedicated to "Soli Deo," the same ruined castle, once the impregnable fortress of the Lascaris family. In all these items it resembles scores of other such hill towns, but the drive there and back is a dream of beauty. In the little Piazza stands a grand tulip tree, and under it the affairs of the nation are discussed by the rank and fashion of Gorbio. From Gorbio there is a way to St. Agnèse, but it is entirely unfit for any but the exceptionally robust.

### Sospello.

This also is a practicable undertaking for delicate folks. It is a drive of fourteen miles each way, and you must take a whole day to accomplish it. Start early (carriage and pair) with your invalid comfortably arranged on his mattress if not equal to sitting up so long. A rest of a couple of hours will be advisable at Sospello for the horses, and the ruined town itself is extremely interesting. The church and the bridge over the Bevera are most picturesque; the latter has a curious tower in its centre. Of all the excursions from Mentone that can be made by carriage I think this is, on the whole, the most charming.

### Castiglione.

If Sospello is considered too fatiguing a drive go to Castiglione instead, five miles short of Sospello. The little town, with its extraordinarily twisted streets, resembles Roccabruna, but the houses are still more unsophisticated, the entrances seeming often mere cavities in the rock, but the roofs are gay with painted tiles. Hare tells us that Castiglione seems inhabited only by old crones and lean fowls. It stands on an arid rock, and is strangely at variance with the smiling landscape around. From here there are several cross roads to enchanted spots, but they are entirely unfitted for invalids. They can only look and long, unfortunately.

### The Corniche to Ventimiglia.

The road from Mentone to Ventimiglia is especially fine. A short drive is to La Mortola. The drivers only ask half an hour's rest before returning, but it is advisable to put up the horses at the little village and spend some hours there. In the first place the fairy gardens of the Villa Mortola must be examined (cards to view to be had at the hotels); it is a veritable paradise. The little village is charmingly situated and most picturesque with its gaily-coloured church. Close by here the road winds round and doubles on itself in a striking manner. Grimaldi and Ciotti are both near, but there is no carriage road. It is necessary to climb a stair-like path.

### Excursions for the Robust.

I think foremost among these I must place St. Agnèse. It is a fatiguing jaunt, but delightful all the same. No way of progression but on one's feet is possible. There are two ways to reach it; the nearest is by the road pats the Borrigo Torrent, but it is a good plan to drive as far as Gorbio, and whilst the carriage is waiting there take the cross path. This will occupy two hours. The little town itself is forty-five minutes from the summit of the mountain, which is 2,510 ft. above sea level. Here stands the remains of a tenth century castle. St. Agnèse is more like Eza than any other spot along the Riviera, but is more barren and gloomy and more inaccessible.

In Old Mentone.

The Gossip Place of Gorbio.

In returning mind not to miss the right path; you will be quite tired enough without adding to your walk. I am sure, however, that you will greatly enjoy your excursion and feel that it is worth all the fatigue. Another place for good walkers to see, is Peille. I bicycled and walked from Nice, but there is, I believe, a much shorter way from Mentone, passing Gorbio, and by this route you can be helped by carriage. Peille is quite a *terra incognita* to most sojourners on the Riviera, so too is St. Agnèse, Eza, Biot, and hundreds of other lonely spots hidden in the mountains. The aspect of Peille as you slowly advance upon it is grand and awe inspiring, not on account of the small cluster of buildings which constitutes the village, but by reason of the somewhat gloomy grandeur of the surroundings and the intense solitude. Peillon may be visited the same day by helping yourself as far as Gorbio by carriage. It will take the whole day, but by visiting these two places you get a good idea of the striking scenery north of the Corniche. I am told that occasionally wolves are seen in these solitudes in the winter, and I can well believe it. The church at St. Agnèse is dedicated to "Notre Dame de la Niege." There is a curious custom and procession there on the festa day which I should much like to have seen. The greatest andholder in the neighbourhood offers to the curé of the village a gilded apple, which he presents (clothed in Court dress) personally; in the good old days this apple was filled with money, but now it is a barren offering. All the feminine population of St. Agnèse join in the procession with veils on their heads and lighted candles in their hands.

### TRAVEL NOTES AND QUERIES.

BRUGES (Palette).—Yes, delightful for sketching, and living is cheap there even now, though not so markedly as a few years since. The place you mean is the Minnewater. The legend is that a girl threw herself from the round tower at the north end of the bridge in despair at separation from her lover, and that her spirit haunts the weird Minnewater.

DINAN (Mars).—Not so cheap as St. Servan, but reasonable pension terms are to be had. A good centre for excursions. For your purpose it would answer well, and so would Morlaix, much further west. Quimper is not so picturesque, but the cathedral makes up for all deficiencies.

SWITZERLAND (Nemo).—Thank you for your appreciation. I understand now your difficulty. Go first to Meiringen, put up at the Hotel Sauvage, and stay a fortnight. If the weather is good you will do a good deal in that time. Then at the end of June move on to Brienz. It is cheaper than Interlaken, and, as far as the town goes, much prettier. The steamers will land you at Interlaken many times a day. Untersen is not a separate town; it is in reality the older part of Interlaken, and far the more picturesque.

WHAT SEASON FOR VENICE? (Tennis Ball).—Decidedly the spring. The autumn is quite as fashionable, but not nearly so agreeable; it is very hot up to the end of September, and the cold makes itself felt very suddenly in October. Venice in April and May is ideal.

NURSING IN UPPER EGYPT (Cairo).—Unfortunately your question was overlooked; the answer should have appeared a fortnight since. Luxor and Assouanare eminently suited for invalids, and at both places there is a resident doctor and nurse at the hotel. The best season for Egypt is from November to the middle or end of March. Yes, there are numerous travellers now that the Nile has been opened up. I do not think there are any nursing homes in Cairo, because the visitors are usually wealthy, and if illness overtakes one not so well off he or she would go to one of the hospitals, of which there are two or three on a basis of partial payment.

FLORENCE (Argosy).—Yes, I think it might suit you. It is a kind of intermediate station, where it is customary to spend a couple of months between wintering on the Riviera and going up to the mountains for the hot weather. It is a very sunny place, rendering it suitable for anæmic people and those suffering from depression. It can, however, be very cold there in early spring. An article will shortly appear dealing with life in Florence.

ROME (Eucalyptus).—See answer to "Argosy." The same may be said of Rome, with this difference, it is warmer, but not so bright. For some reason difficult to explain Rome lacks the brilliancy and gay joyousness of the City of Lilies. 2. Quite possible to live there on the terms you mention. Go to an hotel for a few days while you look about.

*(For Travel Advertisements see Page xix.)*

### Notes and Queries.

**The contents of the Editor's Letter-box have now reached such unwieldy proportions that it has become necessary to establish a hard and fast rule regarding Answers to Correspondents. In future, all questions requiring replies will continue to be answered in this column without any fee. If an answer is required by letter, a fee of half-a-crown must be enclosed with the note containing the enquiry. We are always pleased to help our numerous correspondents to the fullest extent, and we can trust them to sympathise in the overwhelming amount of writing which makes the new rules a necessity.**

**Every communication must be accompanied by the writer's name and address, otherwise it will receive no attention.**

*Disinfection.*

(40) If clothing used by a scarlet fever patient is, after being soaked in carbolic acid 1 in 30, washed with other clothing, will it infect the latter? 2. Is clothing used by a scarlet fever patient free from infection after being soaked in carbolic acid 1 in 30, washed, and boiled?—*Arabella.*

1. Not if all the clothing be boiled for upwards of twenty minutes. 2. Certainly, if boiled long enough to ensure that every portion of the clothing has been subjected to the requisite degree of heat. But it must be remembered that it takes some time for the steam to penetrate into every fold and twist of the material.

*Liver Complaint.*

(41) Could the Scientific Press Company kindly inform me of a hydropathic establishment in the South of England where the waters are good for liver complaint?

Cheltenham is a favourite resort for sufferers from this class of disorder.

*Dispensing.*

(42) Kindly inform me which is the best and cheapest way of learning dispensing and pharmacy. Is there any hospital where I could be taught for a few hours a day in exchange for services given the rest of the day?—*A. B.*

You could only hear of such an appointment by advertisement. Write to the Secretary of the Apothecaries' Hall, Blackfriars, E.C., for particulars of the "assistants" examination.

*University College Hospital.*

(43) Would you kindly let me know when the new matron and staff will take up their duties at University College Hospital?—*A. H.*

Next month we believe.

*L.O.S.*

(44) Could you kindly tell me whether there is any way of obtaining an L.O.S. certificate in six weeks instead of the usual three months?—*Nurse.*

When you have a certificate which satisfies the L.O.S. board of examiners that you have received a sufficient course of lectures and instructions, and have attended not less than twenty labour cases, you may present yourself for examination. Your certificate depends upon your passing that examination. As you are already a nurse perhaps some of the training schools would prepare you in the shorter time. The Matron, British Lying-in Hospital, Endell Street, could advise you.

*District Training.*

(45) Can you tell me of any institute where I could receive, for a few hours daily, some practical instruction in district nursing? I have a slight knowledge of nursing.—*A. S. (S. E.)*

You might write to the Lady Superintendent, Southwark, Newington, and to the Walworth District Nursing Association, Benson Home, 37, West Square, Southwark, S.E., and ask her if she would be willing to make arrangements for you to do as you wish. We must, however, warn you that the task of training probationers is not willingly undertaken by anyone unless the candidates are prepared to remain on the staff.

*Method of Proceeding.*

(46) A short time since the recently-appointed superintendent-nurse at a provincial infirmary had to obtain all her necessary supplies through a junior assistant owing to the absence or indisposition of the matron, there being no assistant matron. The junior assistant conducting herself as though in charge of the institution, who should have been the responsible person in this case? Should not the superintendent nurse or the porter? In the event of a recurrence of the circumstances what course would be the best to follow?—*Charlotte D.*

The responsible person on such occasion is that one deputed by the matron to fulfil her duties, no matter whom she may be. The only course open to the other officers is to do their own allotted work as though the matron herself were present.

*Breach of Contract.*

(47) A Matron writes: Last month I advertised in your valuable paper for a nurse. I had many applications, and finally engaged a nurse to come on the 11th of the month. She asked, would I be willing for her to wait until the 13th, then she could then stay Sunday over with some friends. This I was pleased to do. Since then I have neither heard or seen anything of the nurse. This has put me to the greatest inconvenience, and I have been obliged to advertise again. Can anything be done to stop this kind of thing? I should be glad to have your opinion on the matter.—*Matron.*

You could prosecute her for breach of contract if you cared to go to the trouble and expense.

*Musical Instrument.*

(48) Would you kindly inform me what kind of small musical instrument, easy to learn, would be best for me to begin? 2. Can you give me any information concerning the examination to qualify for a sanitary inspector? I understand nurses can do so.—*Nurse Nina.*

The guitar is easily learnt, and is the best of the small stringed instruments played without a bow. 2. Write to the Secretary, the Sanitary Institute, Margaret Street, W. Nurses are likely to make good sanitary inspectors, but no special advantages are offered them in the examinations.

*Free Homes for Phthisis.*

(49) Would you be kind enough to tell me if there are any free sanatoriums where the open-air treatment for tuberculosis is carried out, and, if there are no free ones, which is the cheapest?—*M. E. B.*

Will you kindly give me the address of a Sanatorium for Phthisis, where the open air treatment is carried out. Patient, a man of 39 years, with one lung affected, could not afford high terms.—*District Nurse.*

See "Sanatoria for Consumptives," by F. R. Walters, M.D., price 10s. 6d. (Swan, Sonnenschein, and Co.) The Royal National Hospital for Consumption, Ventnor, takes patients free, and we believe the treatment is being carried out on a small scale at the North London Hospital for Consumption at Hampstead.

*Medical Charities.*

(50) Would you kindly let me know if there is any medical fund from which the widow of a medical man could obtain assistance? She has been left without anything, as her husband lost his money in a speculation.—*Masseuse.*

Any of the following might help her, if her circumstances warranted: The British Medical Benevolent Fund, 84, Brook Street, Grosvenor Square, W.; the *Lancet* Relief Fund, the Secretary, the *Lancet* Office, Strand, W.C.; the Royal Medical Benevolent College, Office 37, Soho Square, W.; the Society for the Relief of Widows and Orphans of Medical Men; the Universal Beneficent Society, 13, Soho Square, W.

*An Oculist.*

(51) Could you tell me the address of an oculist whose fee would not exceed half a guinea for testing my eyesight for glasses? I cannot afford more yet; do not want to go to a hospital for charity.—*H. E.* [illegible]

We cannot recommend individuals. You might write to the Secretary of the Ophthalmic Hospital, Moorfields, E., explaining the circumstances, and he might possibly give you a letter to one of the specialists attending the hospital, who would see you privately at the fee you name.

*Institutions.*

(52) Will you kindly tell me of the best general hospital in the Midland counties where a young probationer may receive a good training?—*Anxious.*

If you send 2s. to the Manager of the Scientific Press he will send you a copy of the "Nursing Profession: How and Where to Train," in which you will find full particulars.

## Figures and Tables

**Figure f1:**
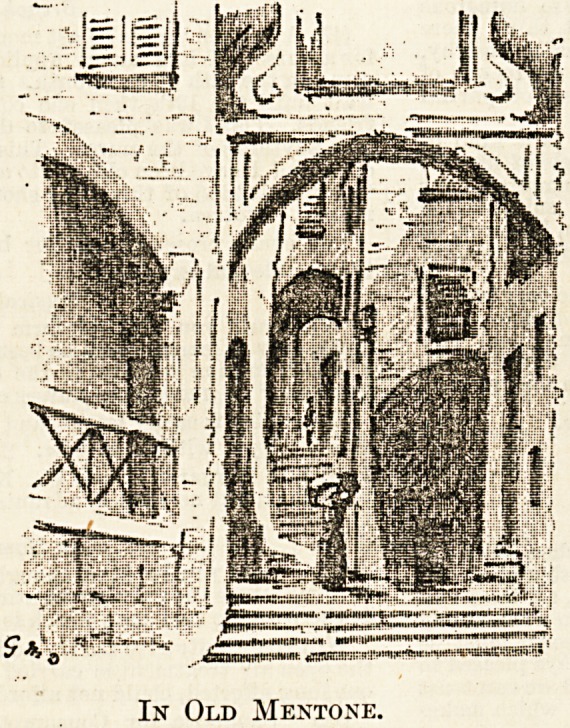


**Figure f2:**